# The potential mechanism of extracellular high mobility group box-1 protein mediated p53 expression in immune dysfunction of T lymphocytes

**DOI:** 10.18632/oncotarget.22913

**Published:** 2017-12-04

**Authors:** Ying-Yi Luan, Min Jia, Hui Zhang, Fu-Jun Zhu, Ning Dong, Yong-Wen Feng, Ming Wu, Ya-Lin Tong, Yong-Ming Yao

**Affiliations:** ^1^ Department of Burns and Plastic Surgery, The 181st Hospital of Chinese PLA, Guilin 541002, People's Republic of China; ^2^ Trauma Research Center, First Hospital Affiliated to the Chinese PLA General Hospital, Beijing 100048, People's Republic of China; ^3^ Department of Critical Care Medicine, The Second People's Hospital of Shenzhen, Shenzhen 518035, People's Republic of China

**Keywords:** high mobility group box-1 protein, p53, p38 mitogen-activated protein kinase, T lymphocytes, immune dysfunction

## Abstract

In the present study, we examined the activity of p53 protein in Jurkat cells treated with high mobility group box-1 protein (HMGB1), thereafter we investigated the mechanism of extracellular HMGB1 mediated p53 expression in immune dysfunction of T lymphocytes. mRNA expression of p53, mdm2, and p21 was determined by Real-time reverse transcription-polymerase chain reaction(RT-PCR). The apoptotic rate of Jurkat cells was analyzed by flow cytometry. Expressions of bcl-2, bax, caspase-3, phosphorylated (p) extracellular signal-regulated kinase (ERK)1/2, ERK1/2, p-p38 mitogen-activated protein kinase (MAPK), p38 MAPK, and p-c-jun amino-terminal kinase (JNK)1/2 and JNK1/2 were simultaneously determined by Western blotting. After treatment with HMGB1 (100 ng/ml or 1000 ng/ml), the proliferative activity of Jurkat cells was significantly decreased, and a low and medium concentration of HMGB1 induced an up-regulation of p53 mRNA, p-p53 and p53 protein expression. Meanwhile, levels of mdm2 and p21 were elevated by incubated with HMGB1 (100 ng/ml) for 24 or 48 hours. Moreover, the proliferation of Jurkat cells in response to HMGB1 (100 ng/ml) in the vector group was significantly depressed. The bax and caspase-3 levels in p53 shRNA-expressed cells treated with HMGB1 (100 ng/ml) was markedly decreased, whereas expression of bcl-2 was obviously enhanced. Among ERK1/2, p38 MAPK and JNK1/2 signaling, only p38 MAPK pathway could be significantly activated by treatment with HMGB1, and the specific inhibitor of p38 MAPK was used, p53 and p-p53 expression induced by HMGB1 were significantly down-regulated. Taken together, our data strongly indicated that HMGB1 might enhance p53 expression, which was associated with both the proliferative activity as well as apoptosis of T cells.

## INTRODUCTION

It has been demonstrated that high mobility group box-1 protein (HMGB1) plays a well-established role as a pro-inflammatory mediator during innate immune response to injury. In the development of sepsis, the T lymphocytes are critically involved in cell-mediated immune response, reproductive activity has remarkably attenuated and the T helper cell (Th)2 immune reaction has become predominant [1, 2]. HMGB1 can regulate T-cell mediated immunity, thus the underlying mechanism has not been fully elucidated.

The p53 protein is a regulative factor of many processes necessary for the proper functioning of cells, and it corresponds to a number of processes associated with its life and death. The p53 protein regulates the repair of cellular DNA and induces apoptosis when the damage of the gene is too serious and it is impossible to repair [3]. Mouse double minute 2 (mdm2) was shown to bind tightly to p53 and inhibit its biochemical activity by a variety of means [4, 5]. It is well known that intrinsic apoptotic pathway is initiated by the activation of p53. p53 can directly modulate the expression of a host of Bcl-2 family proteins which divided into two main groups of proteins: a pro-apoptotic ones such as Bax and anti-apoptotic ones including Bcl-2 [6–8], thereby directly contributing to mitochondrial outer membrane permeabilization, release of cytochrome C and apoptosis. What is more, several signaling pathways have been implicated in regulating the intrinsic apoptotic pathway. Among them, nuclear factor kappa B (NF-κB) and mitogen-activated protein kinase (MAPK) are considered substantial intermediates for induction of apoptosis [9, 10].

It has been documented that intracellular HMGB1 and p53 could interact with each other by a direct binding, the HMGB1/p53 complex affects the cytoplasmic localization of the reciprocal binding partner thereby regulating subsequent levels of autophagy and apoptosis [11, 12]. In contrast to cytokine secretion, HMGB1 redistribution required the p53 tumor suppressor, but not its activator ATM. Moreover, altered HMGB1 expression induced a p53-dependent senescent growth arrest, and the secreted HMGB1 was essential for optimal secretion of interleukin (IL)-6 and tumor necrosis factor (TNF)-α [13]. However, the definite effect of extracellular HMGB1 on p53 activity has not been illustrated. The present study was performed to investigate the effect of extracellular HMGB1 on proliferation of T lymphocytes, and to reveal the potential role of p53 on HMGB1 mediated dysfunction of T lymphocyte proliferation as well as apoptosis. Furthermore, the study was conducted to explore the signal transduction mechanism underlying HMGB1 activated p53 expression associated with MAPK pathways.

## RESULTS

### HMGB1 inhibited the proliferative activity of Jurkat cells

**Figure 1:**
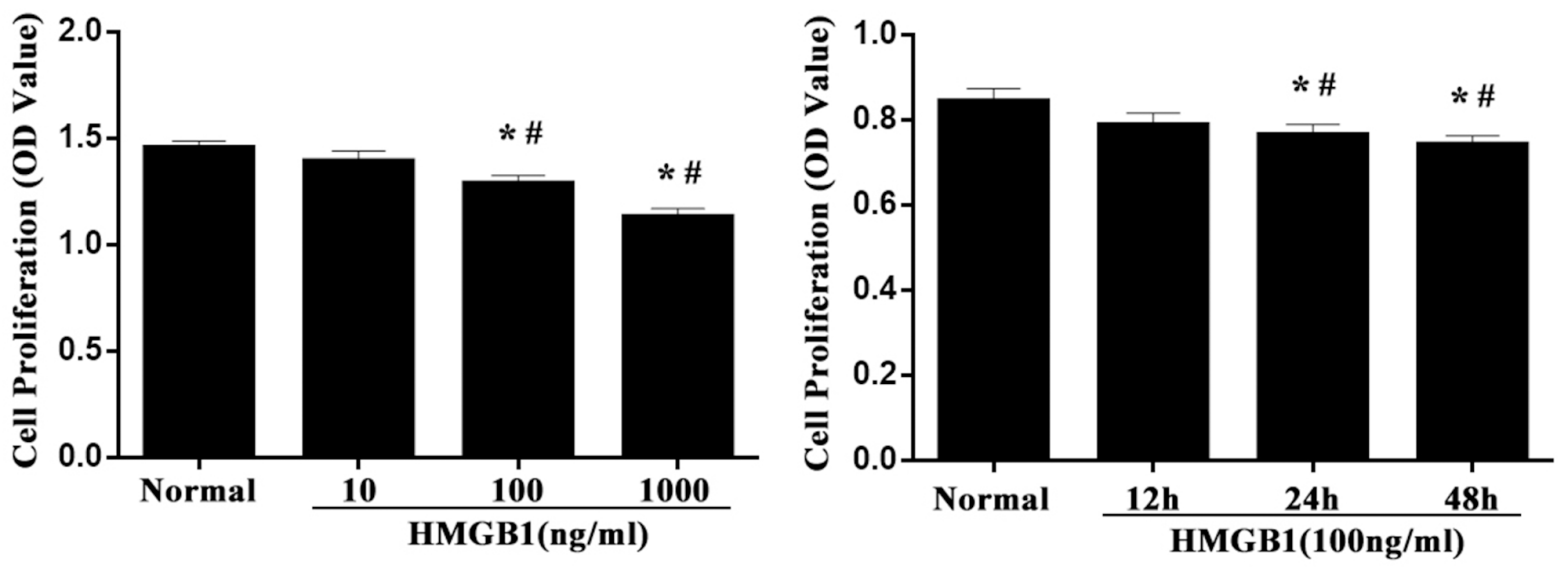
The proliferative activity of Jurkat cells after HMGB1 stimulation Jurkat cells were incubated with HMGB1 (10 ng/ml, 100 ng/ml or 1000 ng/ml) for 24 hours, or incubated with HMGB1 (100 ng/ml) for 12, 24, and 48 hours. CCK-8 was used to measure T-cell proliferative activity. Results of experiments were shown as the mean ± SD. Statistical significance: ^*^P<0.05, compared with the normal group; ^#^*P*<0.05, compared with HMGB1 (100 ng/ml) treated for 12-hour group or HMGB1 (10 ng/ml) treated group.

To investigate the effect of HMGB1 on the proliferative activity of Jurkat cells, cells were analyzed at designated time points. As shown in Figure 1, proliferative activities of Jurkat cells were significantly decreased by incubated with HMGB1 (100 ng/ml) for 24 or 48 hours (*P*<0.05). Treatment with medium and high concentration of HMGB1 (100 ng/ml or 1000 ng/ml) markedly inhibited cell proliferation at 24 hours (all *P*<0.05).

### HMGB1 up-regulated both p53 mRNA and protein levels

**Figure 2:**
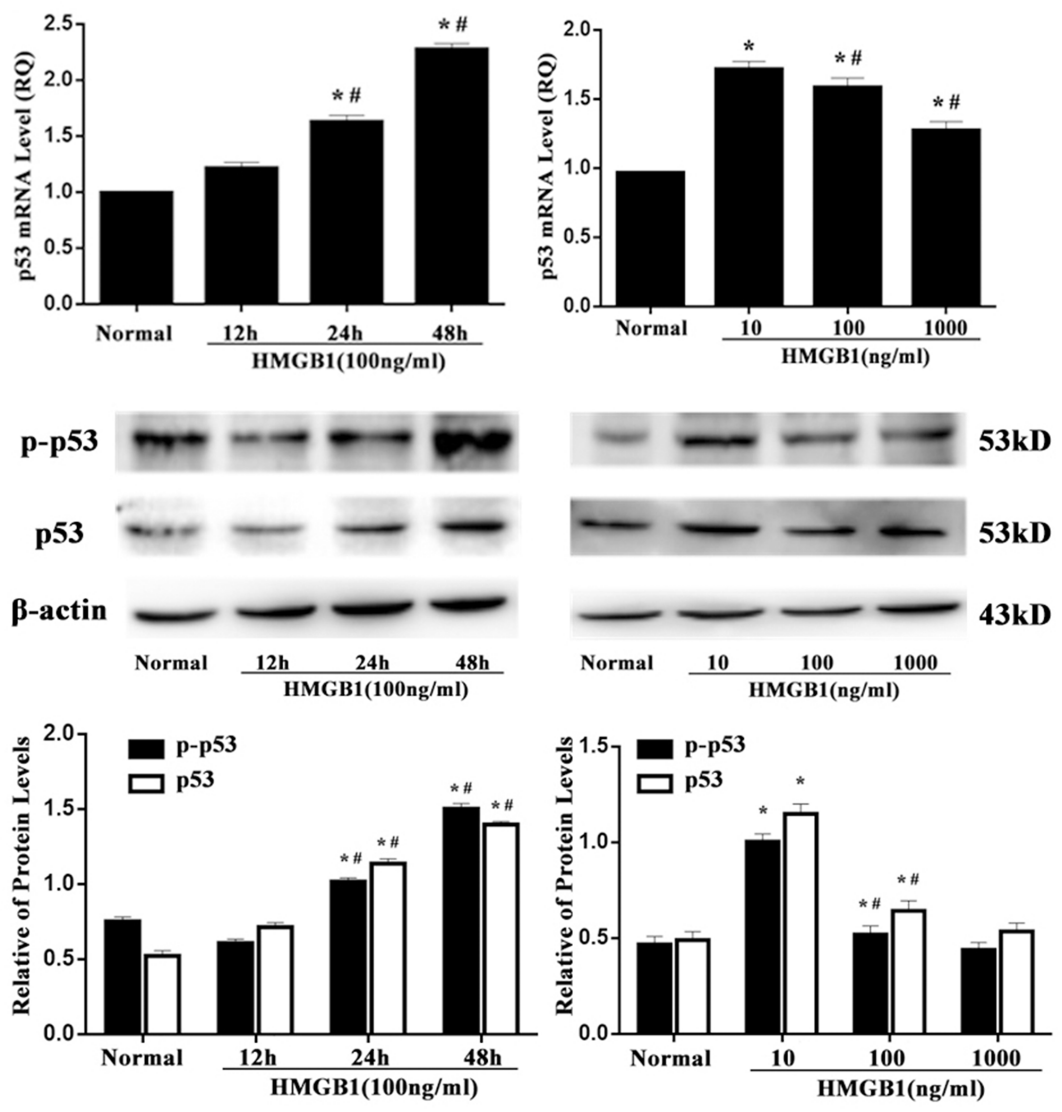
p53 mRNA and protein expressions after HMGB1 stimulation p53 mRNA, phospho-p53 and p53 protein expressions determined by RT-PCR and Western blot analysis in Jurkat cells were significantly up-regulated after 24 and 48 hours of HMGB1 (100 ng/ml) treatment or after 24 hours of HMGB1(10 ng/ml or 100 ng/ml) stimulation. Results of experiments were shown as the mean ± SD. Statistical significance: ^*^P<0.05, compared with the normal group; ^#^*P*<0.05, compared with HMGB1 (100 ng/ml) treated for 12-hour group or HMGB1 (10 ng/ml) treated group.

After 24 and 48 hours of HMGB1 (100 ng/ml) stimulation, p53 mRNA expression was gradually up-regulated. Consistently, phosphorylated p53 and p53 proteins were accumulated (Figure 2). In a dose-dependent manner, a low and medium concentration of HMGB1 induced an increase of p53 mRNA, phospho-p53 and p53 protein expressions. Nevertheless, a high concentration of HMGB1 stimulation did not obviously alter p53 expression.

### HMGB1 increased mRNA and protein levels of mdm2 and p21

**Figure 3:**
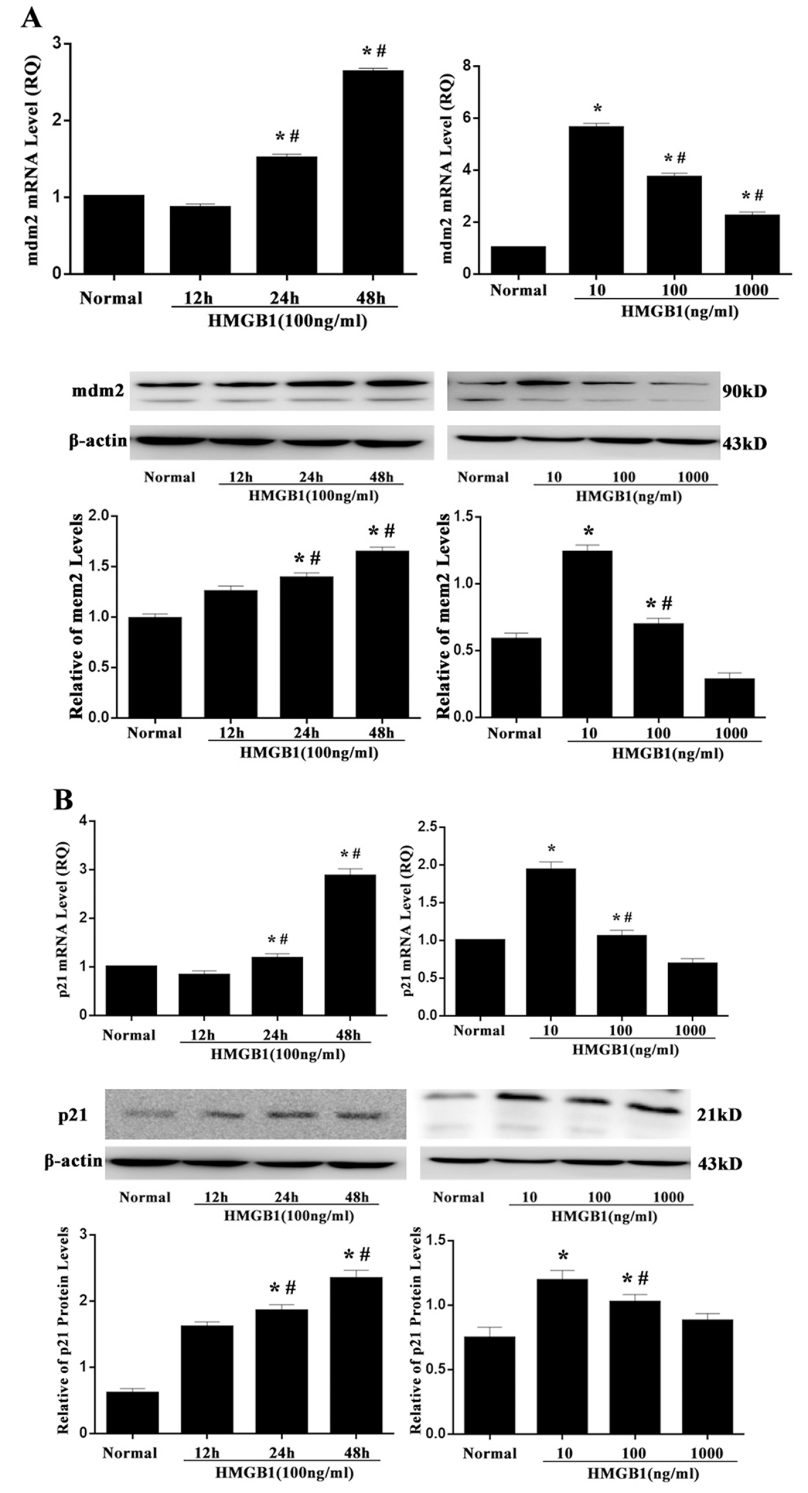
mRNA and protein levels of mdm2 and p21 after treatment with HMGB1 mRNA and protein expressions of mdm2 **(A)** and p21 **(B)** measured by RT-PCR and Western blot analysis in Jurkat cells were markedly enhanced after 24 and 48 hours of HMGB1 (100 ng/ml) treatment or after 24 hours of HMGB1 (10 ng/ml or 100 ng/ml) stimulation. Results of experiments were shown as the mean ± SD. Statistical significance: ^*^P<0.05, compared with the normal group; ^#^*P*<0.05, compared with HMGB1 (100 ng/ml) treated for 12-hour group or HMGB1 (10 ng/ml) treated group.

It is well known that the up-regulated p53 possessed transcription activity, indicating by an elevation of its target gene mRNA expression, including p21 and mdm2. As shown in Figure 3, both mdm2 and p21 expressions were strongly enhanced by incubated with HMGB1 (100 ng/ml) for 24 or 48 hours compared with that in the normal controls (all *P*<0.05). Treatment with a low and medium concentration of HMGB1 (10 ng/ml or 100 ng/ml), the expressions of mdm2 and p21 compared with the normal group, were significantly higher (all *P*<0.05). Furthermore, the effect of HMGB1 on the p21 and mdm2 was consistent with that of p53.

### Effects of p53 on T-cell proliferation and differentiation induced by HMGB1

**Figure 4:**
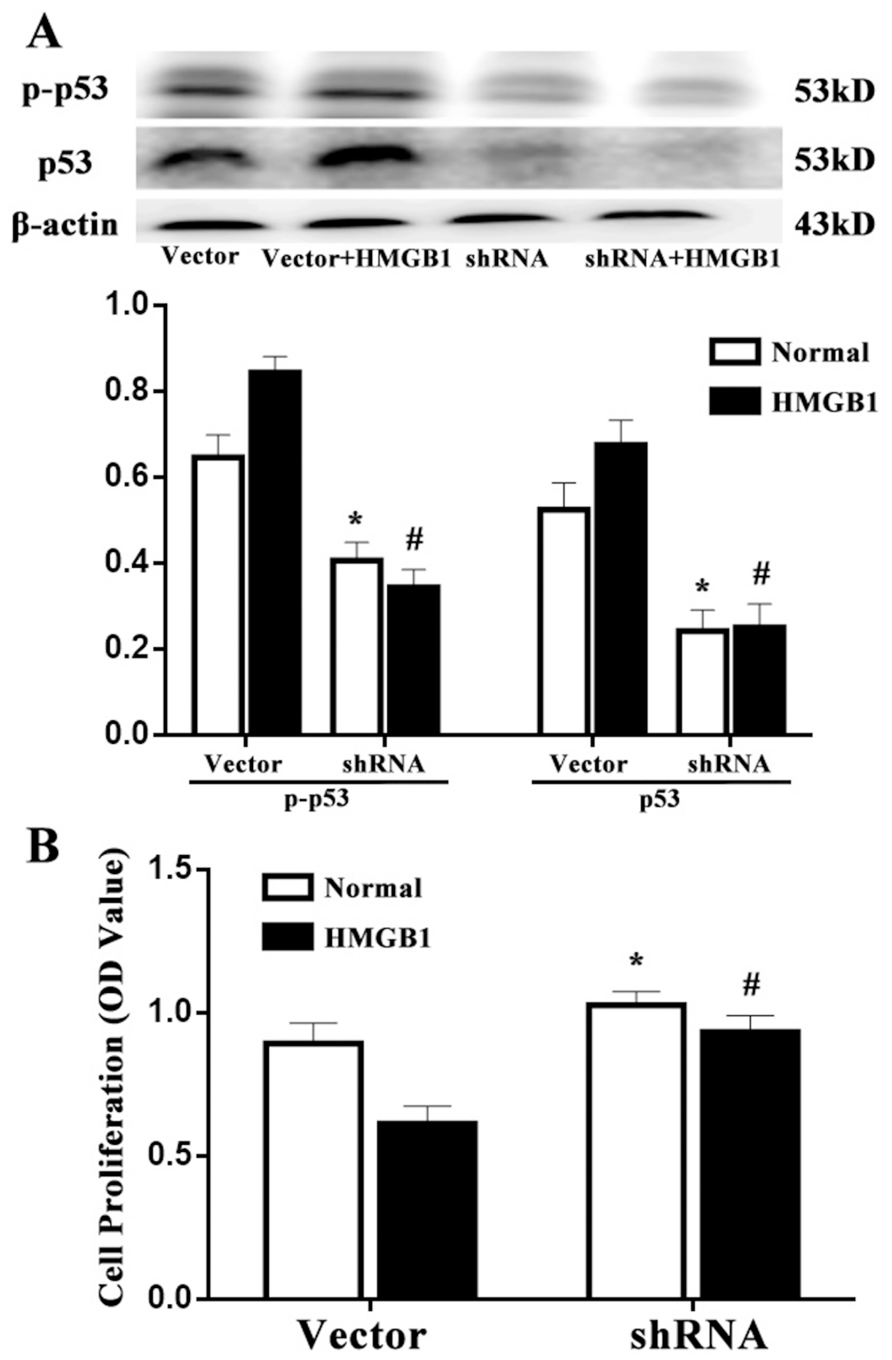
Effects of p53 on T-cell proliferation induced by HMGB1 **(A)** By Western blot analysis, p53 protein level was obviously decreased in p53-shRNA transfected Jurkat cells compared with the vector controls. **(B)** p53-shRNA treatment could enhance T-cell proliferative activity response to HMGB1. CCK-8 was used to measure T-cell proliferative activity. Results of experiments were shown as the mean ± SD. Statistical significance: ^*^P<0.05 as p53 shRNA (HMGB1 untreated) group versus the vector (HMGB1 untreated) group; ^#^*P*<0.05 as p53 shRNA (HMGB1 treated) group versus the vector (HMGB1 treated) group.

p53 gene silenced by shRNA was used in this experiment, concomitantly, the same numbers of vector was served as wild type control. p53 expression was significantly down-regulated in p53-shRNA transfected Jurkat cells compared with the vector controls (Figure 4A, *P*<0.05). As shown in Figure 4B, p53 shRNA and vector expressed cells were constructed and subjected to HMGB1 stimulation. We found that the proliferation of Jurkat cells in response to HMGB1 (100 ng/ml) in the vector group was significantly depressed, but not in p53 shRNA-expressed cells (*P*<0.05).

**Figure 5:**
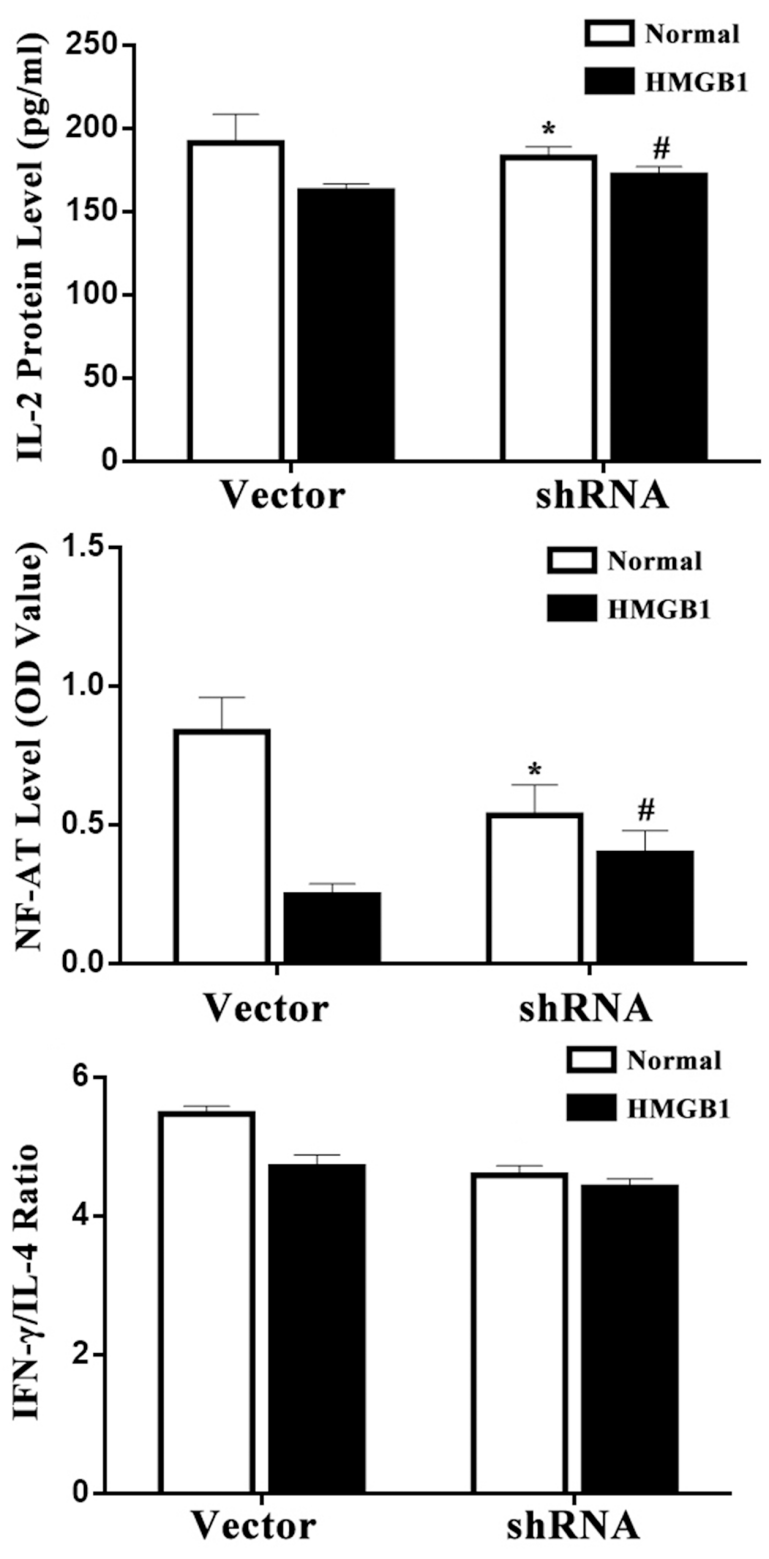
Effects of p53 on T-cell immune function induced by HMGB1 IL-2 level in conditioned media and intranuclear NF-AT activity of T cells in the nuclear cell extract were measured by ELISA. IL-4 levels in culture medium were determined by ELISA to evaluate Th1/Th2 polarization. Treatment with HMGB1 significantly increased IL-2 level and NF-AT activity, but did not markedly decrease IFN-γ/IL-4 ratio in p53 shRNA-expressed cells. Results of experiments were shown as the mean ± SD. Statistical significance: ^*^P<0.05 as p53 shRNA (HMGB1 untreated) group versus the vector (HMGB1 untreated) group; ^#^*P*<0.05 as p53 shRNA (HMGB1 treated) group versus the vector (HMGB1 treated) group.

Further, we performed an experiment to assess NF-AT activation, which increased transcription of cytokine including IL-2. As shown in Figure 5, in the HMGB1 treated vector cells, NF-AT activity showed a dramatic decrement. Meanwhile, cells presented a lower IL-2 production and a shift to Th2 phenotype indicating by a decreased IFN-γ/IL-4 ratio. However, treatment with HMGB1 significantly increased IL-2 expression and NF-AT activity in the p53 silence group, but did not markedly decrease IFN-γ/IL-4 ratio in p53 shRNA-expressed cells (*P*<0.05).

### p53 mediated HMGB1-induced cell apoptosis

**Figure 6:**
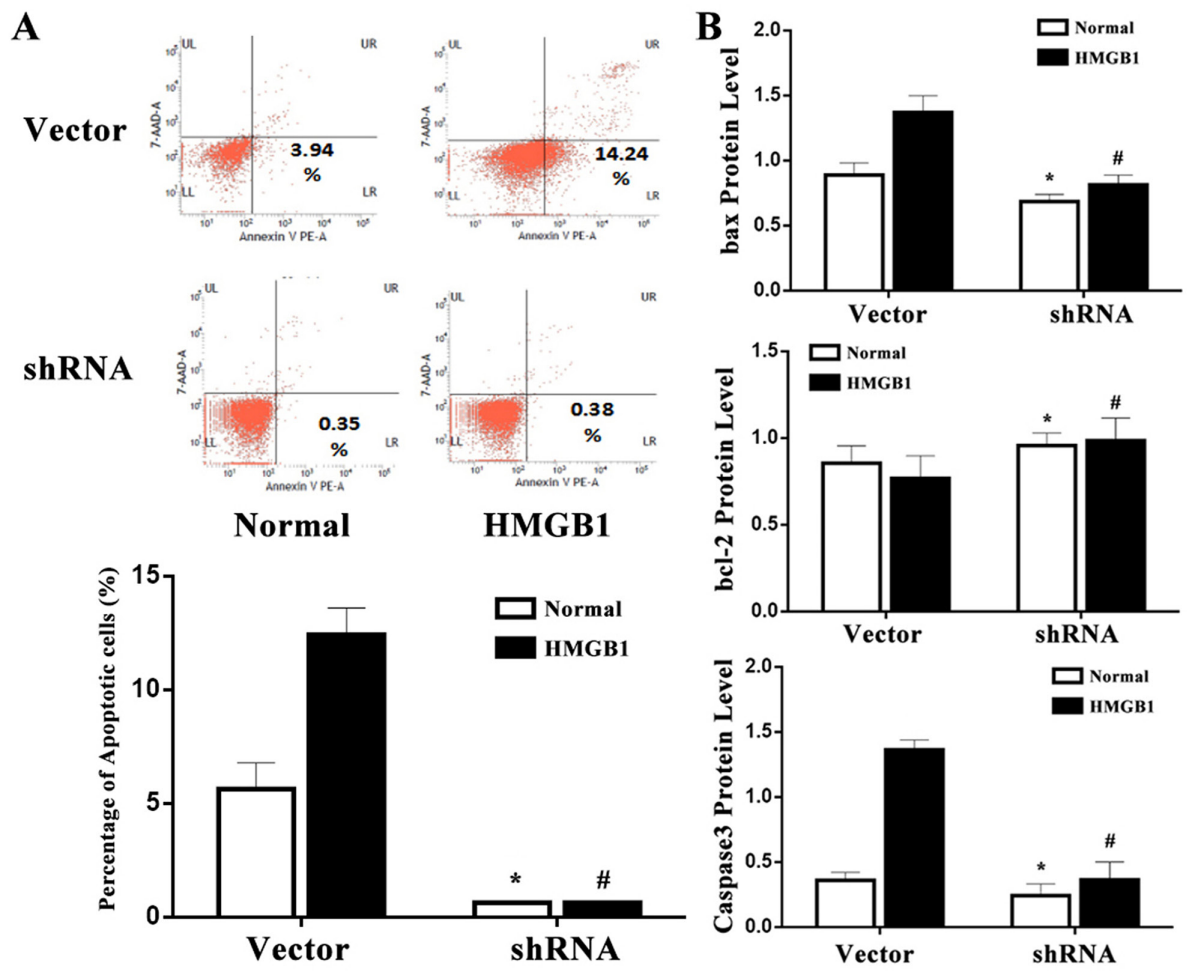
p53 mediated HMGB1-induced cell apoptosis **(A)** Using flow cytometric technique, the apoptotic rate of Jurkat cells was measured. The apoptosis rate of vector-expressed cells after HMGB1 treatment was significantly higher than that of p53 shRNA-expressed cells. **(B)** The protein expression of bcl-2, bax and caspase-3 were determined by Western blot analysis. Results of experiments were shown as the mean ± SD. Statistical significance: ^*^P<0.05 as p53 shRNA (HMGB1 untreated) group versus the vector (HMGB1 untreated) group; ^#^*P*<0.05 as p53 shRNA (HMGB1 treated) group versus the vector (HMGB1 treated) group.

The p53 protein can regulate the repair of cellular DNA and induce apoptosis. As shown in Figure 6, HMGB1 (100 ng/ml) stimulation promoted a significant apoptosis in vector-expressed cells (*P*<0.05), while a less apoptosis was shown in the p53 shRNA-expressed cells. To understand the potential mechanism concerning the involvement of p53 in apoptosis of Jurkat cells, changes in bcl-2, bax, and caspase-3 were analyzed. The bax and caspase-3 levels in vector-expressed cells incubated with HMGB1 (100 ng/ml) were markedly elevated as compared with the normal control, whereas could be decreased when p53 gene was silenced by shRNA (all *P*<0.05, Figure 6). Additionly, change trend in bcl-2 was contrast to bax and caspase-3, bcl-2 expression in p53 shRNA-expressed cells incubated with HMGB1 (100 ng/ml) was markedly higher than that in the vector group (*P*<0.05).

### HMGB1 induced p53 activation was regulated by p38 MAPK

**Figure 7:**
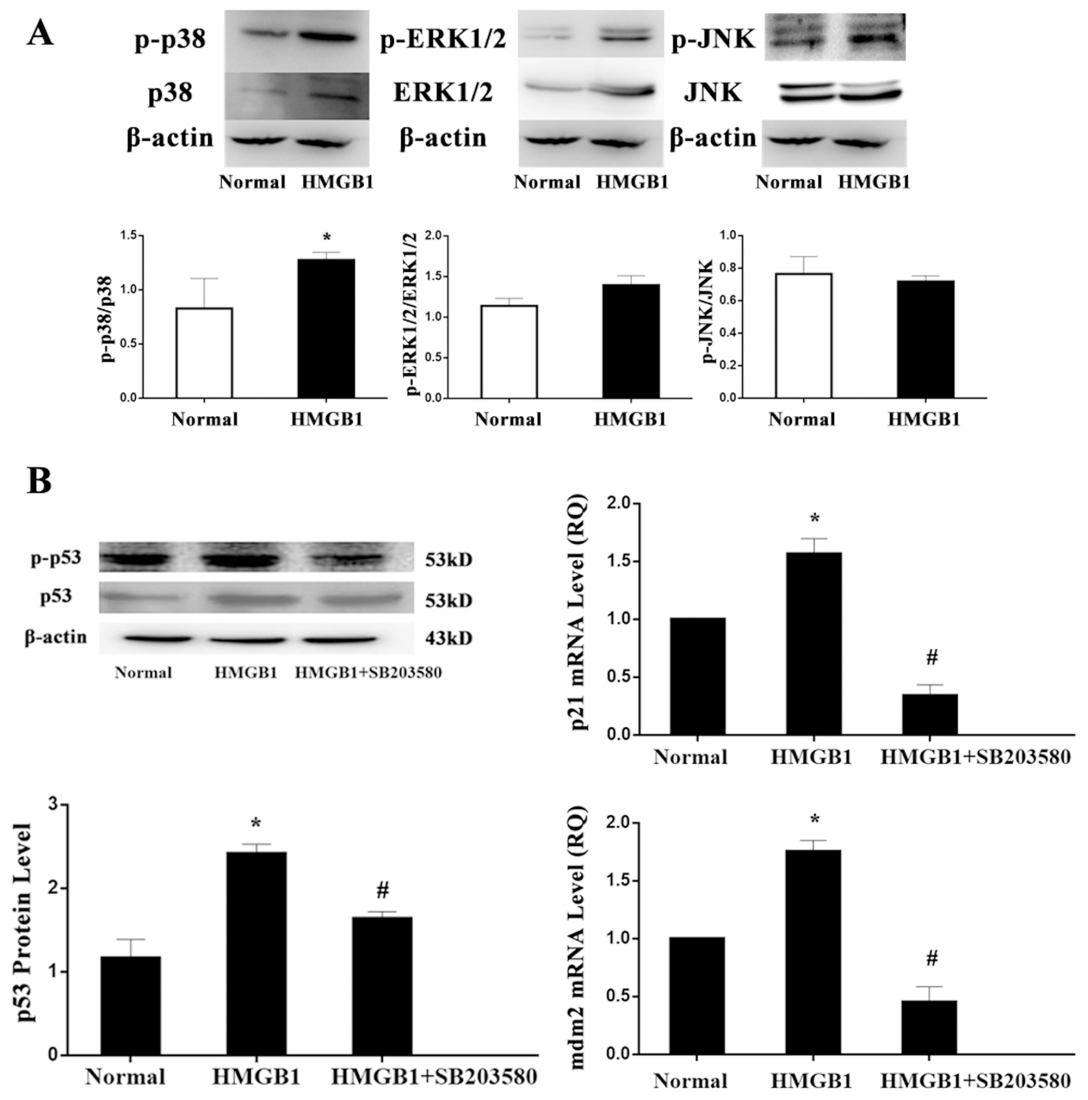
HMGB1 induced p53 activation was regulated by p38 MAPK **(A)** The protein expressions of ERK1/2, p38 MAPK, and JNK1/2 were determined by Western blot analysis. **(B)** Treatment with SB203580, p53, mdm2, and p21 mRNA were markedly down-regulated. Results of experiments were shown as the mean ± SD. Statistical significance: ^*^P<0.05, compared with the normal group; ^#^*P*<0.05, compared with the HMGB1 treated group.

MAPK cascades are highly conserved signaling networks that transduce the signals elicited by physiological and stress stimuli, which mainly includes three signaling pathways, ERK1/2, p38 MAPK, and JNK1/2. In the present experiment, protein levels of ERK1/2, p38 MAPK, and JNK1/2 were determined by Western blot analysis, but among three major MAPK pathways, only p38 MAPK pathway could be significantly activated by the treatment with HMGB1 (Figure 7A). Thereafter the specific inhibitor (SB203580) of p38 MAPK was used, we found that treatment with SB203580, enhanced p53 expression induced by HMGB1 were reversed including mRNA and protein expressions (*P*<0.05; Figure 7). Consistently, its target gene, mdm2 and p21mRNA transcriptions were also down-regulated by the treatment with SB203580 as compared with the HMGB1-stimulated group (all *P*<0.05).

## DISCUSSION

HMGB1, originally identified as a nuclear nonhistone DNA-binding protein, has more recently been shown to act as an extracellular mediator of inflammation [14]. It has been indicated in animal experimentation that the serum levels of HMGB1 are increased at late time points after endotoxin exposure and also that delayed administration of antibody to HMGB1 attenuates endotoxin lethality [13, 14–16]. Several lines of evidence have suggested that HMGB1, acts as a late pro-inflammatory cytokine, at lease in part, is known to play an important role in regulating the function of number of immune cells, such as monocyte, macrophage, dendritic cells (DCs), T lymphocytes, and regulatory T cells (Tregs). During sepsis, There is a growing body of evidence to show that T cells are critically involved in cell-mediated immune response, showing a significant reduction in reproductive activity together with predominant shift to Th2 polarization [17–20]. Experiments *in vitro* showed that the proliferative activities of Jurkat cells were significantly decreased by incubated with HMGB1 (100 ng/ml) for 24 or 48 hours or treated with medium to high concentrations of HMGB1 (100 ng/ml or 1000 ng/ml). However, the interaction between extracellular HMGB1 and T lymphocytes has not been clear, and the effects of extracellular HMGB1 on the function of T cells and its potential mechanism are worth investigating.

In the present study, we revealed the potential role of p53 on extracellular HMGB1 mediated immune dysfunction of proliferation as well as apoptosis in T lymphocytes. By inducing the expression of p21, p53 can inhibit the cell cycle protein (cyclin) and cyclin dependent kinase (cyclin-dependent, kinases, CDKs) expression and interaction, block cell cycle, finally inhibit cell proliferation [21–25]. In the current study, it was found that both protein levels of phospho-p53/p53 and p53 mRNA expression were markedly elevated in Jurkat cells treated with a low and medium concentration of HMGB1, and the results demonstrated that p53 expression was related to extracellular HMGB1, and the activation of p53 induced by HMGB1 was not only through post transcriptional modifications (phosphorylation), but at transcriptional levels. Consequently, treatment with a low and medium concentration of HMGB1 (10 ng/ml or 100 ng/ml), expressions of mdm2 and p21 were noted to be obviously up-regulated. This observation also revealed that HMGB1 did not result in the decrease in expression of mdm2, whereas the trend of mdm2 expression was consistent with p53, indicating that expression of p53 was enhanced and independent of mdm2 in the setting of HMGB1 stimulation. However, the increase in mdm2 mRNA was associated with p53 expression, which further confirmed that up-regulation of p53 might have a normal transcriptional activity. In the current experiment, p53 gene was silenced by shRNA, the same numbers of vector was served as wild type control. The results showed that T-lymphocyte proliferative response to HMGB1 was significantly decreased in the vector group, but it could be increased by treatment with p53 gene silence in Jurkat cells. Levels of IL-2, IFN-γ, IL-4, and intranuclear NF-AT activation were also examined, IL-2 level and NF-AT activity were markedly reduced, but no significant changes were found in IFN-γ/IL-4 ratio in p53 shRNA-expressed cells. The data indicated that p53 might block the suppressive effect of T cell proliferation, secretion of IL-2 induced by HMGB1 without influence on T cell polarization.

As we known, p53 protein can cross into the mitochondria and activate the expression of pro-apoptotic genes including Puma and Bax, and inhibit the expression of anti-apoptotic genes, such as those of the family bcl-2 [26–30]. These proapoptotic proteins, together with the p53 protein, are transported into the mitochondria where they induce an increase in the permeability of mitochondrial membranes and the release of cytochrome c, which connects with the caspase-9 proenzyme. The activation of caspase-9 consequently promotes the caspase-3 proenzyme to the active protease stage, which then adheres to the group of effector caspase [3, 31–36]. In this study, HMGB1 (100 ng/ml) stimulation was noticed to enhance a significant apoptosis in vector-expressed cells, while the apoptosis was markedly decreased in the p53 shRNA-expressed cells. Simultaneously, expressions of bcl-2, bax, and caspase-3 in Jurkat cells were determined, and it was shown that bcl-2 expression was markedly enhanced in p53 shRNA-expressed cells incubated with HMGB1 (100 ng/ml), while bax and caspase-3 were significantly decreased. It was revealed that blockade of p53 could markedly protect against apoptosis of T lymphocytes. Therefore, it was implied that p53 could be involved in the apoptosis of T lymphocytes through activating the intrinsic cell death signaling pathway after HMGB1 stimulation.

Moreover, in the present experiment, protein levels of MAPK signaling pathway, including ERK1/2, p38 MAPK, and JNK1/2 were detected, whereas only p38 MAPK pathway could be significantly activated by the treatment with HMGB1. Thus, the specific inhibitor (SB203580) of p38 MAPK was used in the experiment, we found that treatment with SB203580, enhanced p53 mRNA and protein expression induced by HMGB1 were obviously reversed. Consistently, its target gene, mdm2 and p21 mRNA transcription expressions were also inhibited by the treatment with SB203580. Our data suggested that the alteration in mdm2 and p21 was in contrast to p53, and blockade of p38 MAPK, the transcriptional up-regulatory role and phosphorylated modification of p53 could be inhibited by HMGB1. Nevertheless, this study has some limitations. In the present experiment, the shRNA was used to knockdown the p53 expression, but no up-regulation of p53 expression was employed in Jurkat cells. In a further study, the possible effect of up-regulation of p53 expression on HMGB1-medited T immune response should be included.

In conclusion, we reported that HMGB1 was closely associated with p53 activity in Jurkat cells, and p53 protein could influence both the proliferation as well as apoptosis of T lymphocytes. The proapoptotic proteins, together with the p53 protein, activated the intrinsic cell death signaling pathway. Bcl-2 superfamily members could be activated through increasing expression of pro-apoptotic protein bax and reducing the expression of anti-apoptotic protein bcl-2. In addition, HMGB1 affected p53 via activating the p38 MAPK signaling pathway. Taken together, these findings suggest that treatment with HMGB1 appears to enhance p53 expression, and signals mediated by p38 MAPK might be critically involved in this process, thereby contributing to the development of immune dysfunction of T lymphocytes.

## MATERIALS AND METHODS

### Medium and reagents

Rat anti-mouse bcl-2 and Bim antibody were purchased from Santa Cruz Biotechnology, Santa Cruz, CA. Recombinant HMGB1 was purchased from R&D System, Minneapolis, MI. Rat anti-mouse p53, phosphorylated p53, bcl-2 and Bax antibody were purchased from Cell Signaling Technology, Beverly, MA. MAPK family antibody sampler kit and phospho-MAPK family antibody sampler kit were purchased from Cell Signaling Technology, Beverly, MA. Annexin V- fluorescein isothiocyante (FITC) was purchased from BD, San Diego, CA. The p38 MAPK inhibitor (SB203580) was purchased from Selleck Chemicals, Houston, TX. Enzyme-linked immunosorbent assay (ELISA) kits of IL-12, IL-2, IL-4, interferon (IFN)-γ, and TNF-α were purchased from Biosource, Worcester, MA. Nuclear extract and nuclear factor of activated T cell (NF-AT) assay kits were purchased from Active Motif, Carlsbad, CA.

### CCK-8 assay

Jurkat cells were purchased from cell bank of Chinese Academy of Sciences, and were plated into 96-well flat bottom plates at 2×10^5^ cells/well, incubated with HMGB1 (10 ng/ml, 100 ng/ml, 1000 ng/ml) in a medium containing 10% fetal calf serum (FCS) at 37°C in 5% CO_2_ in humidified air for 24 hours, or incubated with HMGB1 (100 ng/ml) in a medium containing 10%FCS at 37°C in 5% CO_2_ in humidified air for 12, 24, and 48 hours, respectively(39). Thereafter, 10 μl CCK-8 solution was added to each well, the optical density of T-cell proliferative activity was measured by the use of a microplate reader(39).

### Flow cytometric analysis

The apoptotic rate of Jurkat cells was measured by flow cytometry, 5 μl of freshly prepared 7-AAD and phycoerythrin (PE)-Annexin V was added to each sample, and negative control was designed. Finally, cells were fixed in 1% formaldehyde/phosphate buffered saline (PBS) and analyzed by FACSCalibur using CellQust software.

### Extraction of total RNA and RT-PCR

Total RNA was extracted from Jurkat cells using TRIZOL kits as we described previously [37–39, 40]. The concentration of purified total RNA was determined spectrophotometrically at 260 nm. p53, mdm2 and p21 mRNA expression in cells was quantified by SYBR Green two-step, real-time RT-PCR. After removal of potentially contaminating DNA with DNase I, 1 μg of total RNA from each sample was used for RT with an oligo dT and a Superscript II to generate first-strand cDNA. The mRNA level of β-actin was also designed as an internal control for each sample. Primers for p53 were forward 5’- CAGCAGATGACGGAGGTTGT-3’ and reverse 5’- TCATCCAAATACTCCACACGC-3’. Primers for p21 were forward 5’-CAGTGGAACTTCGACTTTGTCA-3’ and reverse 5’-GCACAAGGG TACAAGACAGTG-3’. Primers for mdm2 were forward 5’- CAGTAGCAGTGAATCTACAGGGA-3’ and reverse 5’-CTGATCCAACAAATC ACCTGAAT-3’. For β-actin, we used the following primers: forward 5’- ATTGGCAATGAGCGGTTCCG-3’, reverse 5’-AGGGCAGTGATCTCCTTCTG-3’. The thermal cycling conditions were as follows: 94°C for 5 minutes, followed by 35 cycles of denaturation, annealing and amplification (94°C for 30 seconds, 57°C for 45 seconds, 72°C for 1 minute), and a final extension period of 7 minutes at 72°C. All samples were run in quadruplicates(39).

### RNA interference

Small interference RNA to p53 (shRNA) was synthesized by Obio Technology Corp., Shanghai, China. To knock-down p53 expression, Jurkat cells were infected with recombinant lentiviruses that carry the p53 shRNA. The DNA target sequence for p53 shRNA was 5’-CCACTGGATGGAGAATATT-3’. shRNA transfection was performed according to the manufacture's instruction in Lentiviral Vector Particle. Three days later, western blot was used to check the efficiency of knock-down for P53.

### Western blotting

50 μg of total proteins/sample were loaded on 10% Tris-HCl-sodium dodecyl sulfate-polyacrylamide gel, and the products were electro-transferred to an immobilon polyvinylidene difluoride membrane. After blocking with 10% skim milk overnight at 4°C, the membrane was incubated for 6 hours at room temperature with anti- bcl-2, bax, caspase-3, p-ERK1/2, ERK1/2, p-p38 MAPK, p38 MAPK, p-JNK1/2, and JNK1/2 antibodies, followed by peroxidase-labelled affinity secondary antibody at a dilution of 1:5000 for 1 hour at room temperature(40). After three washings, the membrane was determined by using the ECL plus chemiluminescence kit.

### Cytokine and NF-AT activity measurements with ELISA

The nuclear cell extract was prepared. Levels of IL-2, IL-4, IFN-γ, and the active form of NF-AT contained in nuclei were measured with ELISA(39).

### Statistical analysis

Data was represented as mean ± standard deviation (SD). Data sets were examined by one-way ANOVA, and individual group means were then compared with Student paired t test(39). All statistical tests were two sided and a P value of 0.05 or less was considered to indicate statistical significance.
